# Gemcitabine-Loaded Microbeads for Transarterial Chemoembolization of Rabbit Renal Tumor Monitored by ^18^F-FDG Positron Emission Tomography/X-Ray Computed Tomography Imaging

**DOI:** 10.3390/pharmaceutics16121609

**Published:** 2024-12-17

**Authors:** Xiaoli Zhang, Tingting Li, Jindong Tong, Meihong Zhou, Zi Wang, Xingdang Liu, Wei Lu, Jingjing Lou, Qingtong Yi

**Affiliations:** 1 Department of Urology and Department of Nuclear Medicine, Shanghai Pudong Hospital, Fudan University Pudong Medical Center, Shanghai 201399, China; xiaolizhang22@m.fudan.edu.cn (X.Z.); 18918355023@163.com (M.Z.); 18379882822@163.com (Z.W.); xingdliu@fudan.edu.cn (X.L.); 2 Quzhou Fudan Institute, Quzhou 324002, China; 21111030042@m.fudan.edu.cn (T.L.); wlu@fudan.edu.cn (W.L.); 3 Key Laboratory of Smart Drug Delivery, Ministry of Education & State Key Laboratory of Molecular Engineering of Polymers, School of Pharmacy & Minhang Hospital, Fudan University, Shanghai 201203, China; 4 Department of Vascular Surgery, Shanghai Pudong Hospital, Fudan University Pudong Medical Center & Shanghai Key Laboratory of Vascular Lesions Regulation and Remodeling, Shanghai 201399, China; jindong1220@163.com

**Keywords:** drug-eluting beads (DEBs), transarterial chemoembolization (TACE), gemcitabine, 2-deoxy-2-[(18)F]fluoro-D-glucose (^18^F-FDG), positron emission tomography/X-ray computed tomography (PET/CT), renal cell carcinoma (RCC)

## Abstract

Background/Objectives: The purpose of this study was to develop the gemcitabine-loaded drug-eluting beads (G-DEBs) for transarterial chemoembolization (TACE) in rabbit renal tumors and to evaluate their antitumor effect using 2-deoxy-2-[(18)F]fluoro-D-glucose positron emission tomography/X-ray computed tomography (^18^F-FDG PET/CT). Methods: DEBs were prepared by polyvinyl alcohol-based macromer crosslinked with *N*-acryl tyrosine and *N*,*N*′-methylenebis(acrylamide). Gemcitabine was loaded through ion change to obtain G-DEBs. Their particle size and drug release profile were characterized. VX2 tumors were implanted in the right kidney of rabbits to establish the renal tumor model. The tumor-bearing rabbits received pre-scan by ^18^F-FDG PET/CT, followed by targeted transarterial injection of G-DEBs under digital subtraction angiography (DSA) guidance. The rabbits received another ^18^F-FDG PET/CT scan 10 or 14 days after the treatment. The therapeutic effect was further validated by histopathological analysis of the dissected tumors. Results: The average particle size of the microspheres was 58.06 ± 0.50 µm, and the polydisperse index was 0.26 ± 0.002. The maximum loading rate of G-DEBs was 18.09 ± 0.35%, with almost 100% encapsulation efficiency. Within 24 h, GEM was eluted from G-DEBs rapidly and completely, and more than 20% was released in different media. DSA illustrated that G-DEBs were delivered to rabbit renal tumors. Compared with the untreated control group with increased tumor volume and intense ^18^F -FDG uptake, the G-DEBs group showed significant reductions in tumor volume and maximum standard uptake value (SUV_max_) 10 or 14 days after the treatment. Histopathological analysis confirmed that the proliferating area of tumor cells was significantly reduced in the G-DEBs group. Conclusions: Our results demonstrated that G-DEBs are effective in TACE treatment of rabbit VX2 renal tumors, and ^18^F-FDG PET/CT provides a non-invasive imaging modality to monitor the antitumor effects of TACE in renal tumors.

## 1. Introduction

Cancer is a major global health issue. Renal cell carcinoma (RCC) accounts for 80%–90% of malignant kidney tumors and shows a rising incidence rate year by year [1,2,3]. Over the past few decades, despite the continuous development of cancer diagnostic and therapeutic strategies, RCC remains one of the most lethal malignancies of the urinary system [4,5].

The primary treatment for RCC is surgery [3]. Unfortunately, some advanced RCC patients lose the opportunity for surgery at the time of diagnosis and must be considered for chemotherapy. Conventional chemotherapeutic drugs have poor water solubility, low bioavailability, and cytotoxicity to normal cells throughout the body after systemic distribution, thus limiting the drug dosage [6]. Transarterial embolization (TAE) as an interventional treatment technique has been applied to patients with advanced renal cancer, especially RCC [7]. TAE achieves an antitumor effect by blocking the tumor’s blood supply, inducing tumor necrosis or shrinkage [8]. Nevertheless, recanalization of the embolized vessel, tumor neovascularization, and the formation of collateral circulation after TAE lead to tumor recurrence and metastasis, reducing the therapeutic effect of TAE [9].

To overcome the limitation of TAE therapy, transarterial chemoembolization (TACE) has been developed to improve the curative effect by combining embolic agents with chemotherapeutic drugs. In such a case, the chemotherapeutic agents are intraarterially delivered to the tumor site with the embolic agents and released locally, thus reducing their systemic side effects [10]. Specifically, the embolic agents play a pivotal role since they serve as drug carriers to control drug release. Among these agents, drug-eluting beads (DEBs) are optimal options for the bead–drug combinations. DEBs are mainly prepared by polyvinyl alcohol (PVA) crosslinked with 2-acrylamido-2-methyl propane sulfonate. DEBs are capable of drug loading via an ion exchange mechanism as well as continuous drug release in physiological fluids [11] Furthermore, in order to deliver DEBs directly to the target lesion while minimizing embolization of normal parenchyma, superselective TACE was utilized under the digital subtraction angiography (DSA) to accurately identify the tumor-feeding arteries [12]. DEBs effectively loading doxorubicin have been widely used for TACE therapy of liver cancer. Clinical studies have demonstrated that DEB-TACE exhibited superior therapeutic efficiency against hepatocellular carcinoma (HCC) with a better tumor response, reduced adverse effects, and enhanced survival rates [13,14]. Despite success in liver cancer treatment, DEBs for the RCC have been barely reported [15].

Positron emission tomography/computed tomography (PET/CT) has been widely applied to the diagnosis, staging, treatment, and prognosis prediction of various tumors [16]. PET/CT mainly relies on morphology and enhancement patterns to distinguish between benign and malignant tumors, while 2-deoxy-2-[(18)F]fluoro-D-glucose (^18^F-FDG) PET/CT provides metabolic information [17]. Molecular imaging techniques with ^18^F-FDG PET/CT can quantitatively assess glucose utilization in tumor tissues, thus achieving diagnostic purposes [18,19,20]. The standardized uptake value (SUV), defined as the ratio of radioactivity per mL of tissue to the injected dose of radioactivity per kg of patient weight, is the most commonly used indicator for evaluating therapeutic response in FDG-PET clinical practice [21]. Although ^18^F-FDG PET/CT has been used for the assessment of renal cancer and metastasis of RCC [22], the ability of ^18^F-FDG PET/CT to monitor the therapeutic effect of TACE in the RCC has not been reported.

In this study, we developed DEBs using PVA-based macromer crosslinked with *N*-acryl tyrosine and *N*,*N*′-methylenebis(acrylamide) to load gemcitabine (G-DEBs). Gemcitabine is widely used to treat various types of renal cancer in humans [23]. G-DEBs were superselected to the renal tumor-feeding arteries by DSA. The effectiveness of G-DEBs TACE therapy has been evaluated in an experimentally induced rabbit renal tumor model. Our results have demonstrated that ^18^F-FDG PET/CT can non-invasively monitor the antitumor effects of G-DEBs delivered via TACE in rabbits bearing renal tumors.

## 2. Materials and Methods

### 2.1. Materials

All of the chemicals and reagents were purchased from Energy Chemical (Shanghai, China), Aladdin (Shanghai, China), Sigma-Aldrich Chemical (Rahway, NJ, USA). The rabbit VX2 anaplastic squamous cell carcinoma cell line was obtained from American Type Culture Collection (Manassas, VA, USA). Ki67/MKI67 antibody was purchased from Novus Biologicals (Littleton, CO, USA). The terminal-deoxynucleotidyl transferase-mediated nick end labeling (TUNEL) apoptosis detection kit was purchased from Yeasen Biotechnology Co., Ltd. (Shanghai, China). All New Zealand white rabbits (2–3 kg, 3–4 months, male) were provided by Jiaganshengwu Technology Co., Ltd. (Shanghai, China). Zoletil^®^50 was acquired from Virbac (Carros, France).

### 2.2. Preparation of Microspheres

The preparation of microspheres was conducted by inverse suspension polymerization following our previously reported procedure [24]. In brief, the PVA-based macromer was firstly synthesized by the acid-catalyzed reaction of *N*-acryloyl-aminoacetaldehyde dimethylacetal (NAAADA, 400 mg) with the PVA solution (7.5%, *m*/*m*, 10 mL). Then, the macromer solution (2 mL, 100 mg PVA-NAAADA) was mixed evenly with *N*-acryl tyrosine (NAT, 120 mg), *N*,*N*′-methylenebis(acrylamide) (MBA, 40 mg), and potassium persulfate (KPS, 13 mg) in aqueous solution as a water phase. Liquid paraffin (8 mL) containing 0.12 g of Span 80 was prepared as an oil phase. The water phase was added to the oil phase with stirring for 10 min to form an emulsion. *N*,*N*,*N*′,*N*′-tetramethylenediamine (TMEDA) aqueous solution (10%, *v*/*v*, 200 μL) was added to the emulsion dropwise to catalyze the polymerization crosslinking reaction and solidification to form microspheres. The solution was stirred in a 37 °C water bath for 12 h under a nitrogen atmosphere for protection. The microspheres were collected by centrifugation at 5000 rpm for 5 min and washed twice with ethyl acetate, absolute ethanol, and ultrapure water sequentially. Then, the precipitation was wet-seized with 70 μm- and 40 μm-sized mesh screens sequentially.

### 2.3. Morphological Characterization of Microspheres

The microsphere suspension was added into the optimal cutting temperature compound (OCT, Sakura Finetek, Tokyo, Japan) and frozen at −80 °C for cryosectioning into 10 μm-thick slices. A small drop of microsphere suspension and the slices of microspheres were placed on the scanning electron microscopy (SEM) stage, dried, and gold-plated using an ion sputtering coater subsequently. The morphologies of the microspheres and cross-sections were observed by SEM (Ultra 55, Zeiss, Oberkochen, Germany). The size distribution was measured by a Malvern Matersizer 3000 (Worcester, UK).

### 2.4. Drug Loading and Encapsulation Efficiency of G-DEBs

The G-DEBs were prepared by adding gemcitabine (GEM) solution (1 mL) at different concentrations to 1 mL of the DEBs solution (10 mg/mL) under stirring. At a predetermined time, 100 μL of the supernatant was withdrawn for analysis of GEM concentration using a UV–vis spectrophotometer (UV-2401PC, Shimadzu, Kyoto, Japan) at 265 nm. Once the adsorption of GEM reached a steady state, the concentration of the residual GEM in the solution was measured. The drug loading rate and encapsulation efficiency of the DEBs were calculated using the following equations:

Loading⁢ rate⁢ %=mGEM−Vsupernatant×CsupernatantmDEBs+(mGEM−Vsupernatant×Csupernatant)×100%

Encapsulation⁢ efficiency⁢ %=mGEM−Vsupernatant×CsupernatantmGEM×100%

where *m*_GEM_ and *m_DEBs_* represent the mass of GEM and DEBs, respectively. *V_supernatant_* and *C*_supernatant_ indicate the volume of the supernatant and the concentration of GEM in the supernatant, respectively.

### 2.5. Determination of Eluting Capacity and Drug Release

The elution of GEM from the G-DEBs was measured using a previously reported method [23]. Briefly, the G-DEBs (10 mg) were added to 10 mL of phosphate-buffered saline (PBS) at pH 7.4, initiating a sustained elution process in a 37 °C shaking incubator at 150 rpm. Every 2 h, a 3 mL of supernatant release medium was withdrawn, and the concentration of GEM was determined by a UV–vis spectrophotometer. Simultaneously, an equal volume of fresh medium was added to maintain a consistent overall volume of solution.

For the GEM release study, G-DEBs (10 mg) were added to PBS (pH 6.5 and 7.4) in the presence or absence of 10% fetal bovine serum (FBS) at 37 °C in a shaking incubator at 100 rpm. After a predetermined period, 1 mL of the release medium was sampled and replaced with the same volume of the fresh medium. The concentration of GEM in the release medium was evaluated using a UV–vis spectrophotometer. The GEM cumulative release was calculated using the equations below [25]:

Cumulative⁢ release⁢ %=∑i=1n−1Ci×V0+Cn×VmGEM×100%

where *m*_GEM_ represents the total mass of GEM. *C_i_* and *C_n_* indicate the concentration of the GEM solution withdrawn at the time *n* or *i*. *V*_0_ indicates the volume of the withdrawn release medium. *V* stands for the total volume of the release medium, and *n* refers to the number of times of sampling.

### 2.6. In Vitro Antitumor Effect of G-DEBs

A cytotoxicity assay was performed to test the antitumor effect of the drugs in vitro. VX2 cells were cultured in MEM medium supplemented with 10% fetal bovine serum (FBS) and 1% penicillin/streptomycin at 37 °C with 5% CO_2_. VX2 cells were incubated into 96-well plates at a density of 2 × 10^5^ cells/mL for 24 h before the experiment. The cells were incubated with GEM and G-DEBs at concentrations ranging from 0.01 to 100 µM for 24 h and 72 h, respectively. In each well, 10 µL of CCK-8 reagent was added, followed by incubation in the dark at 37 °C for 90 min. Absorbance was measured at 450 nm. For each group, each well was repeated three times to obtain the average value. The control group included untreated cells in complete medium. The experiment was independently repeated three times.

### 2.7. Establishment of the VX2 Renal Tumor Model in Rabbits

All rabbits were anesthetized by Zoletil^®^50 (0.2 mL/kg) via intravenous injection through an indwelling catheter placed in the marginal ear vein. Under general anesthesia, VX2 tumors were harvested from donor rabbits through surgical procedures, which were prepared by injection with VX2 tumor cell suspension into the right thigh muscle [26]. The tumors were then cut into pieces of ~1 mm^3^ using scissors. After anesthesia, the rabbits were positioned in the left lateral decubitus position and fixed on the operating table, where the live tumor fragments were implanted into the right kidney of the rabbit [27]. Two weeks later, when the sizes of the implanted tumor reached 10–20 mm in diameter as detected by ^18^F-FDG PET/CT, the rabbits were randomly assigned for corresponding experimentation.

### 2.8. ^18^F-FDG PET/CT Imaging Acquisition and Analysis

All animals were fasted for at least 12 h before ^18^F-FDG PET/CT imaging. ^18^F-FDG (12 MBq/kg) was administered via the marginal ear vein with a total volume of approximately 1.5 mL. At approximately 60 min after the administration, the rabbits received an intravenous injection of Zoletil^®^50 (0.18 mL/kg) and were in a face-up position using a custom-made fixation device for scanning. All scans were acquired using the NeuWise Pro PET/CT system (Shanghai, China) with a 2 min acquisition time per bed position. Before the PET scan, CT was performed using a multi-detector scanner, with parameters including 120 kV, 400 mA, a 0.6 s rotation time, and a pitch of 1.2, in the same anatomical position. The maximum standardized uptake value (SUV_max_) of the tumor was obtained for each rabbit across multiple planes.

### 2.9. Arterial Embolization of Rabbit Renal Tumor and Validation

After anesthesia by Zoletil^®^50 (0.2 mL/kg), the skin of the right inguinal region of the rabbit was disinfected. A longitudinal incision of 1–2 cm was made along the arterial route. The right femoral artery was bluntly dissected using glass dissecting needles to avoid damage to the femoral vein and nerves. The femoral artery of the rabbit was punctured using an 18-gauge sheath to establish arterial access. A 1.8-F microcatheter (Terumo, Tokyo, Japan) and coaxial micro-guide wire were then inserted through the sheath, utilizing digital subtraction angiography (DSA) (Azurion 3 M15, Philips Medical Systems Nederland B. V., Hamburg, Germany) to guide their approach to the right renal artery and tumor-associated feeding arteries, assisted by the injected iodinated contrast media during the angiographic series. Following the infusion of microspheres into the renal tumor via the microcatheter, arteriography was performed to confirm the complete resolution of hypervascularity in the tumor. On the following day, the rabbit was sacrificed, and the kidney with the tumor was harvested and fixed with 4% paraformaldehyde for hematoxylin-eosin (H&E) staining. The H&E image was captured by a slide scanner (Slideview VS200, Olympus, Tokyo, Japan).

### 2.10. In Vivo Antitumor Therapy

Rabbits bearing the VX2 tumor in the kidney were randomly divided into three groups (*n* = 3): the untreated group (Control), the group receiving transarterial infusion of DEBs (TAE), and the group receiving transarterial infusion of G-DEBs (TACE). The rabbits were fasted for 12 h prior to surgery. After anesthesia, DEBs without GEM or G-DEBs (100 mg/mL) were injected into the renal tumor through a microcatheter following the guidance of DSA described above. After the embolization, the general condition of the rabbits, including feeding, diarrhea, urination, and other adverse reactions, was monitored daily. ^18^F-FDG PET/CT scans were performed one day before treatment and 10 or 14 days post-treatment, respectively.

### 2.11. Histological Analysis

On day 14 post-treatment, all rabbits in each group were euthanized. The renal tumor, as well as the heart, liver, spleen, lung, and normal kidney tissues were harvested. The collected samples were fixed in 4% paraformaldehyde for more than 24 h, embedded in paraffin, sectioned, and stained with H&E. Tumor proliferative cells were detected by immunofluorescence staining using the mouse anti-rabbit Ki67 antibody (1:500) followed by goat anti-mouse Alexa Fluor 594 secondary antibody (1:200). Apoptosis of tumor cells was detected by immunofluorescence staining using the TUNEL apoptosis detection kit. The cell nuclei were counterstained with 4′,6-diamidino-2-phenylindole (DAPI). The tissue samples stained with H&E and immunofluorescence were examined by a slide scanner (Slideview VS200, Olympus, Tokyo, Japan) and confocal laser scanning microscopy (LSM710, Zeiss, Oberkochen, Germany), respectively.

### 2.12. Statistical Analysis

Statistical analysis was performed using Graphpad Prism 9.0. All results were presented as mean ± SD. Statistical significance was determined by two-way analysis of variance (ANOVA) with Tukey’s multiple comparisons post hoc test.

## 3. Results

### 3.1. Preparation and Characterization of DEBs

**Figure 1:**
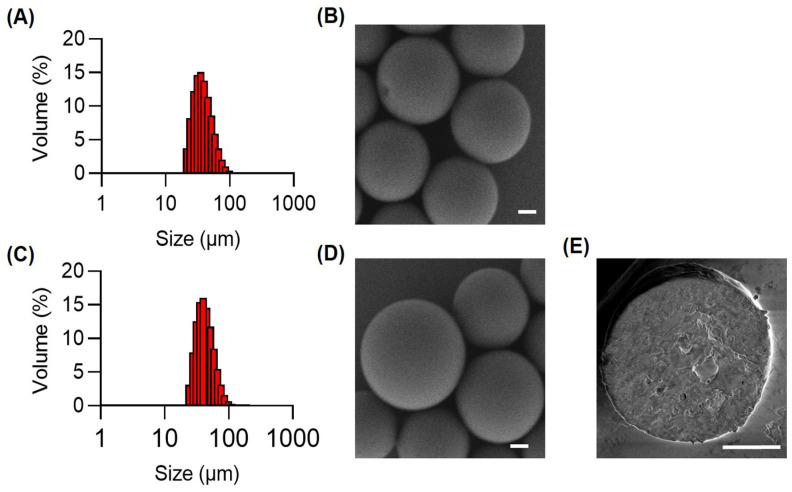
Characterization of DEBs. Size distribution (**A**) and scanning electron micrograph (SEM) (**B**) of DEBs. Size distribution (**C**) and SEM (**D**) of G-DEBs. (**E**) SEM of the cross-section of G-DEBs. Bar, 10 μm.

The average particle size of the microspheres was 58.06 ± 0.50 µm, with a polydisperse index (PDI) of 0.26 ± 0.002 (Figure 1A). SEM image revealed that the prepared microspheres were round in shape, smooth in surface, and uniform in size (Figure 1B). GEM was loaded via ion exchange with sodium ions on the carboxylic group of tyrosine within the microspheres. After drug loading, the microspheres retained their round and uniform morphology with a similar size distribution, showing no significant changes compared with the DEBs (Figure 1C,D). The SEM of the cross-section of the microsphere depicted the interior of the microsphere without obvious holes (Figure 1E).

**Figure 2:**
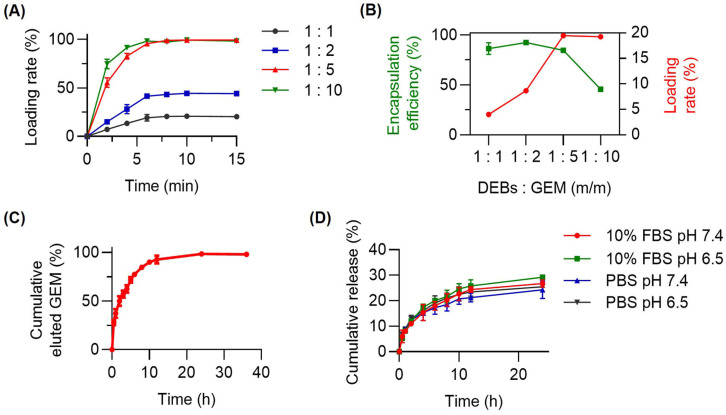
Drug loading and release profiles of DEBs. (**A**) Gemcitabine (GEM) loaded by G-DEBs over time at different mass ratios of DEBs to GEM. Data are presented as mean ± SD (*n* = 3). (**B**) GEM encapsulation (green) or loading (red) efficiency of G-DEBs over different mass ratios. Data are presented as mean ± SD (*n* = 3). (**C**) Percentage of the cumulatively eluted GEM from G-DEBs over time in pH 7.4 PBS. Data are presented as mean ± SD (*n* = 3). (**D**) The cumulative drug release of G-DEBs in pH 6.5 and pH 7.4 PBS with and without 10% FBS. Data are presented as mean ± SD (*n* = 3).

Further characterization revealed that GEM was quickly absorbed by the DEBs at various mass ratios of the DEBs to GEM, reaching a stable state within 10 min (Figure 2A). The results for the encapsulation efficiency and drug loading rate of the G-DEBs demonstrated that the mass ratio of 2:1 (DEBs:GEM) allowed for a maximum drug loading rate of 18.09 ± 0.35% with nearly 100% encapsulation efficiency (Figure 2B). The drug-eluting curve showed that the GEM absorbed into the DEBs was quickly and completely eluted from G-DEBs within 24 h (Figure 2C), facilitated by frequent replacement with bulk fresh medium. The cumulative release curve showed that GEM released over 20% within 24 h in various media (Figure 2D). Collectively, the DEBs were round and uniform, with the property of gemcitabine being easily loaded, completely eluted, and sustained-released, which meets the requirements for in vivo applications.

### 3.2. In Vitro Antitumor Effect of G-DEBs

**Figure 3:**
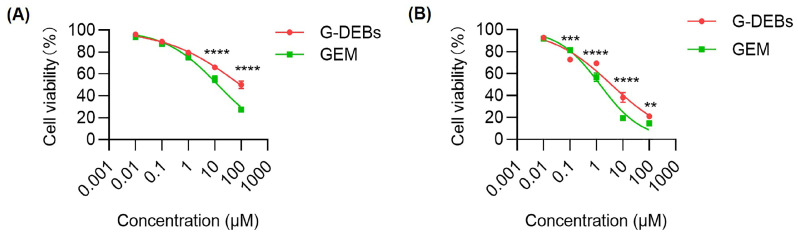
The viability of VX2 tumor cells incubated with GEM and G-DEBs at different concentrations for 24 h (**A**) and 72 h (**B**). Data are presented as mean ± SD (*n* = 3). ** *p* < 0.01, *** *p* < 0.001, **** *p* < 0.0001 between the compared groups. Two-way ANOVA with Tukey’s post hoc test was used for statistical significance.

The antitumor effect of G-DEBs was evaluated in vitro using the CCK-8 assay. The antitumor effect of free GEM or G-DEBs on VX2 cells was concentration and time-dependent. Within the incubation time of 24 h, the antitumor effect of the free GEM group was significantly stronger than G-DEBS at a concentration of 10 μM or above (Figure 3A). The IC_50_ of GEM was calculated to be 13.24 μM, whereas the IC_50_ of G-DEBs was 93.94 μM, possibly attributed to the slow or incomplete release of GEM from G-DEBs within 24 h. When the incubation time was extended to 72 h, the IC_50_ values of free GEM and G-DEBs were both decreased, resulting in 1.37 and 3.67 μM, respectively (Figure 3B).

### 3.3. Embolization of Rabbit Bearing VX2 Renal Tumor Under DSA by G-DEBs

**Figure 4:**
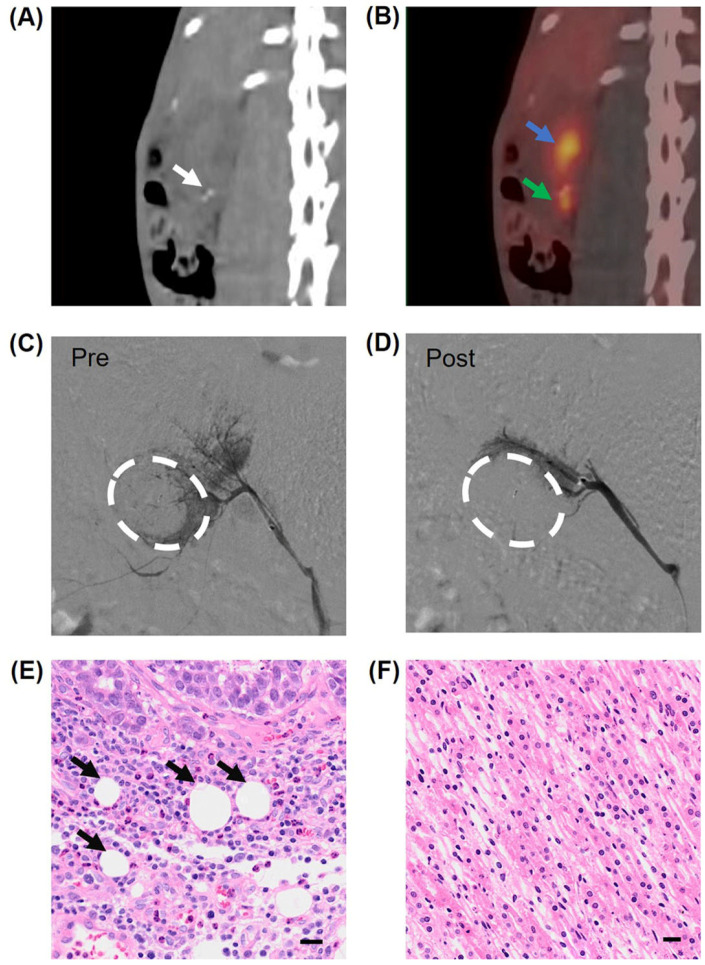
Embolization of rabbit renal tumor with G-DEBs under the guidance of DSA. (**A**,**B**) CT image (**A**) and the corresponding ^18^F-FDG PET/CT image (**B**) of rabbit bearing orthotopic VX2 renal tumor. White arrow, two pieces of micro-guide wires adjacent to the VX2 tumor tissue implanted. Green arrow, the VX2 tumor with positive signals of ^18^F-FDG. Blue arrow, renal pelvis. (**C**,**D**) DSA imaging of VX2 renal tumor before (**C**) and after (**D**) intraarterial infusion of G-DEBs. White dotted circles, tumor. (**E**,**F**) Microscopic images of tumor (**E**) and adjacent kidney tissue (**F**) stained with H&E one day after the embolization. Black arrows, G-DEBs. Bar, 20 µm.

Herein, we established the VX2 renal tumor model in rabbits and used PET/CT to evaluate the growth of tumors with ^18^F-FDG. To verify the location of the tumor, two pieces of micro-guide wires were implanted adjacent to the VX2 tumor piece in the rabbit kidney. The CT image depicted the two white dots of high density, confirming the location of the two pieces of wire (Figure 4A, white arrow). This position was consistent with the region of the positive signals of ^18^F-FDG, demonstrating the implanted renal tumor (Figure 4B, green arrow). Under the guidance of DSA, the renal arteries and blood vessels of tumors were visualized by injecting iodinated contrast media during angiographic series acquisition, allowing for the identification of and access to the feeding arteries of the tumor via a microcatheter (Figure 4C). Then, the G-DEBs were directly injected into the feeding arteries of the renal tumor via the microcatheter, and DSA imaging confirmed that the blood vessels of the tumor were successfully cut off by the G-DEBs (Figure 4D). To further validate the efficient embolization of the renal tumor, the rabbit was euthanized one day post-embolization. Histological analysis by H&E staining confirmed that the G-DEBs were deposited in the tumor (Figure 4E), whereas there was no obvious necrosis or inflammation in adjacent normal kidney tissue (Figure 4F). These results confirmed that the G-DEBs were accurately delivered to the renal tumor with the assistance of DSA.

### 3.4. Enhanced Therapeutic Efficacy of TACE in Rabbits Bearing VX2 Renal Tumor

#### 3.4.1. Enhanced Therapeutic Efficacy Monitored by ^18^F-FDG PET/CT

**Figure 5:**
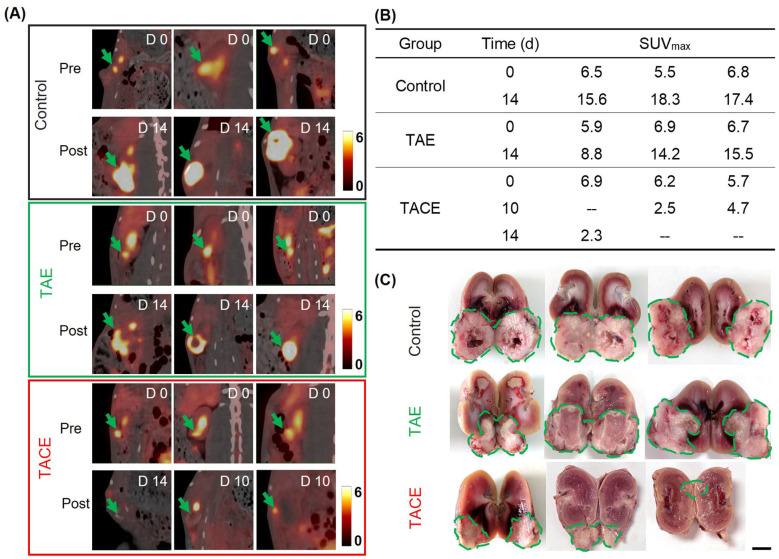
Antitumor effect following G-DEB embolization monitored by ^18^F-FDG PET/CT imaging. (**A**) ^18^F-FDG PET/CT images of rabbits bearing VX2 renal tumor before (Pre) and 10 or 14 days after transarterial infusion of DEBs (TAE) and G-DEBs (TACE) and without treatment (Control) (*n* = 3). Green arrows, tumor. (**B**) SUV_max_ values of ^18^F-FDG in tumors before (0) and 10 or 14 days after different treatments in (**A**). --, no ^18^F FDG PET/CT imaging acquisition. (**C**) Photographs of the resected tumors of each group 14 days following the treatment in (**A**). Bar, 1 cm. Green dotted circles, tumor.

The growth of renal tumors in rabbits was evaluated with ^18^F-FDG PET/CT. The ^18^F-FDG PET/CT imaging result demonstrated the positive signals of ^18^F-FDG uptake by renal tumors, showing the average SUV_max_ of approximately 7 in nine tumor-bearing rabbits (Figure 5A). Without treatment, the average SUV_max_ was increased to 17.10 after 14 days. Comparatively, at 14 days after transarterial injection of DEBs, the average SUV_max_ of ^18^F-FDG in the tumor was 12.83, demonstrating that the embolization of the DEBs slightly delayed tumor growth. By contrast, the G-DEBs significantly inhibited the growth of tumors, with SUV_max_ values of 2.50 and 4.70 at 10 days post-embolization and 2.30 at 14 days post-embolization. Of note, in the TACE group, at 10 and 14 days after transarterial injection of G-DEBs, the SUV_max_ values of 2.50 and 2.30 in the tumor of the two rabbits were close to those of their adjacent normal parenchyma tissue (2.40 and 2.50), respectively (Figure 5A,B). At day 14 post-injection, all rabbits were euthanized, and the kidneys with tumors were resected. The sizes of tumors resected from each treatment group confirmed that the growth of the tumors was significantly inhibited following TACE (Figure 5C), consistent with the result of ^18^F-FDG PET/CT imaging.

#### 3.4.2. Enhanced Therapeutic Efficacy Confirmed by Histological Analysis

**Figure 6:**
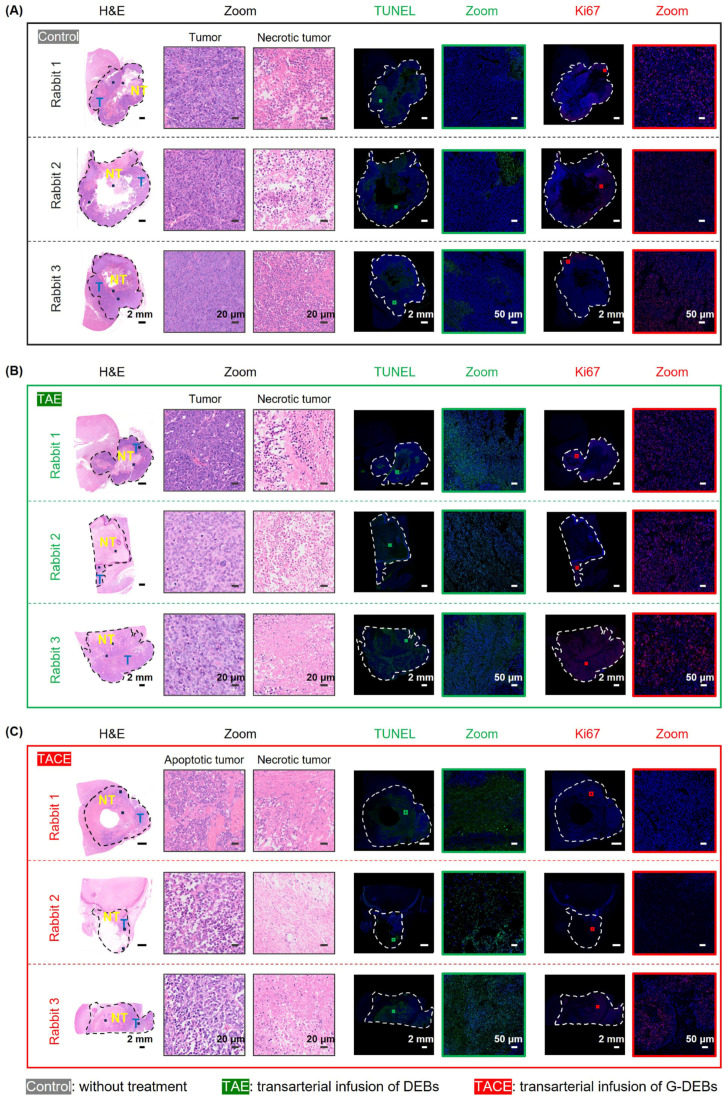
Histological analysis of antitumor effect following G-DEB embolization. (**A**–**C**) Micrographs of H&E staining, TUNEL, and Ki67 immunofluorescence of the renal tumors after transarterial infusion of (**A**) DEBs and (**B**) G-DEBs (TACE) and (**C**) without treatment. In H&E images, the tumor regions are separated from the normal kidney tissues by green dotted lines. T, tumor. NT, necrotic tumor. The box area of tumors in the images is enlarged and presented on the right. In immunofluorescence images, the tumor regions are separated from the normal kidney tissues by white dotted circles.

To analyze the proliferation, apoptosis, and necrosis of the dissected tumor following the above treatment presented in Figure 5C, the tumor samples were sectioned for H&E staining, TUNEL, and Ki67 immunofluorescence staining, respectively. H&E staining revealed various degrees of tissue necrosis in all treatment groups. In the control group, although nuclear pyknosis and necrosis appeared inside the tumor due to insufficient blood supply to the center of the large tumor, the largest area of tumor cells was packed with large and irregular nuclei (Figure 6A). Comparatively, the area of tumor in the TAE group was smaller, while the largest degree of necrosis appeared in the TACE group. For TUNEL staining, the largest green-stained area (specific staining for apoptosis and non-specific staining for necrosis) was observed in the TACE group among all three groups, suggesting maximal apoptosis in this group. Moreover, the expression of Ki67 was assessed as a marker of tumor proliferation. In the control group, the largest number of Ki67-positive cells was observed, indicating that the tumor maintained good proliferative activity. By contrast, the Ki67-positive cells were significantly reduced in the TACE group (Figure 6). Notably, the results of immunofluorescence staining demonstrated that the Ki67-positive cells were barely seen in the samples of the two rabbits in the TACE group (Figure 6C, Rabbits 1 and 2), whereas the TUNEL-positive area of the two rabbits was the largest among all three groups. This result was in line with the data of ^18^F-FDG PET/CT imaging. Collectively, these results confirmed the enhanced antitumor effect of TACE with G-DEBs and the feasibility of ^18^F-FDG PET/CT imaging for accurately monitoring tumor responses.

**Figure 7:**
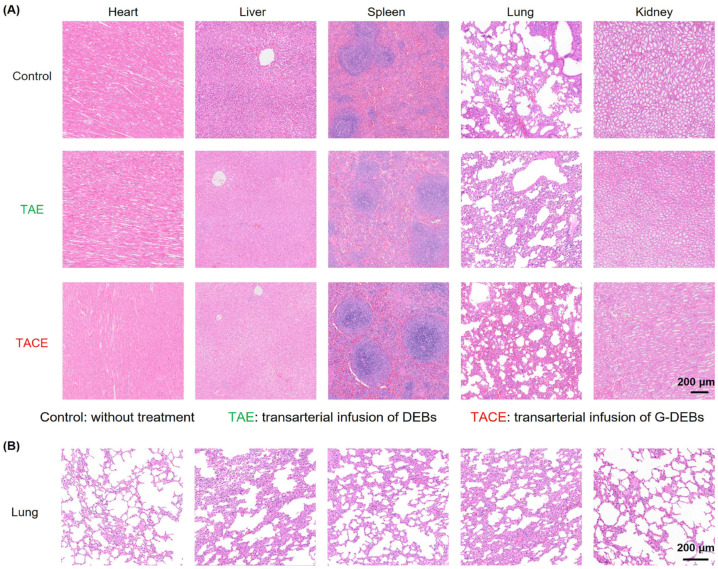
Micrographs of the major organs of rabbits with H&E staining. (**A**) Representative micrographs of H&E staining of the major organs 14 days after transarterial infusion of DEBs (TAE) and G-DEBs (TACE) and without treatment (Control). (**B**) Microscopic images of the lung tissue stained with H&E after the embolization of DEB or G-DEBs.

Moreover, H&E staining images of major organs in each group revealed that there were no obvious changes in the major organs following TAE or TACE treatment with DEBs or G-DEBs, respectively (Figure 7A). Further, no microbeads were observed in H&E staining images of the lung, suggesting accurate embolization under the guidance of DSA without potential off-target embolization (Figure 7B). In summary, TACE with G-DEBs generated a prominent antitumor effect towards VX2 renal tumors without showing obvious toxicity.

## 4. Discussion

TACE is frequently used in HCC. With the development of interventional techniques and embolic agents, TACE has recently been developed for the treatment of RCC [10]. Gemcitabine is one of the most effective chemotherapeutic agents to treat a variety of tumors, including RCC [28]. Generally, gemcitabine is applied through intravenous administration and activated through a series of phosphorylation. However, gemcitabine has a short half-life and is rapidly metabolized for deamination in plasma. Therefore, repeated administration is required in clinical treatment, which leads to severe myelosuppression, hepatotoxicity, and renal toxicity [29]. Chemical modification of drugs (pro-drug) and drug encapsulation using nanocarriers (nano-drug) are two interesting research directions to improve their accumulation in tumor tissues [30]. Compared to pro-drugs and gemcitabine-loaded nano-drugs, superselective DEB-TACE through a locoregional drug delivery approach under DSA allows the DEBs to be carried by blood flow directly to the tumor, where gemcitabine is specifically released into the tumor from the surface of DEBs. This results in ~100% drug bioavailability in the tumor, completely avoiding the off-targeting effect in other normal tissues [31]. DEB-TACE could ultimately improve the therapeutic efficacy and reduce adverse effects, showing great potential for clinical application in the future. In our study, the G-DEBs were prepared for intraarterial delivery of GEM to renal tumors. Compared to other groups, G-DEBs effectively inhibited the growth of VX2 tumors, significantly reducing the maximum diameter of the rabbit VX2 renal tumors and successfully inducing tumor necrosis and apoptosis in the target area. Histopathological analysis demonstrated that the tumor necrosis rate increased after TACE treatment, while no significant changes were observed in other major organs. The results indicated that TACE with G-DEBs specifically enhanced the accumulation of cytotoxic drugs in renal tumors and reduced their distribution in healthy tissues, thus decreasing systemic toxicity.

Furthermore, compared with surgical resection of RCC, DEB is an interventional treatment as an embolization option for unresectable RCC. DEB-TACE can not only embolize tumor-supplying arteries but also sustainedly release the antitumor drug specific to the tumor. In a clinical study, after TACE with doxorubicin-loaded callispheres DEBs, the objective response rate at 1, 3, and 6 months (47.1%, 29.0%, and 23.1%, respectively), median progression-free survival (21.4 months), and median overall survival (24.6 months) in RCC patients demonstrated good disease control rates and prognosis [15]. G-DEBs have been used in the clinical treatment of lung cancer, which is safe and feasible [32]. Our results showed that G-DEBs can effectively and safely embolize the renal tumor-feeding arteries in rabbits. Therefore, TACE combined with G-DEBs represents an effective and promising approach for the treatment of RCC.

As an emerging molecular imaging technology, ^18^F-FDG PET/CT is increasingly recognized for its application in tumor diagnosis [33]. ^18^F-FDG PET/CT has been used to evaluate the therapeutic effect of TACE, but it is mainly used for liver tumors. There is a paucity of relevant reports on RCC. In a case report, ^18^F-FDG uptake in the RCC of patients showed that SUV_max_ and SUV_mean_ were 6.1 and 4.2, respectively. However, there was a lack of SUV values after TACE treatment [34]. In our study, the results showed that the average SUV_max_ of nine tumor-bearing rabbits was about 7. The tumor SUV_max_ in the untreated group was 17.10 after 14 days. In contrast, the SUV_max_ values of TACE were 2.50 and 4.70 at 10 days and 2.30 at 14 days. The ^18^F-FDG PET/CT imaging results were consistent with the results of pathological examination. These results showed the potential of ^18^F-FDG PET/CT for accurately evaluating the efficacy of TACE in RCC. SUV_max_ could be a good indicator of tumor size.

However, this study has some limitations. It has been reported that SUV_max_ can predict the prognosis of RCC patients [35]. In our study, the tumor-bearing rabbits were euthanized after 10 or 14 days of TACE treatment. The ^18^F-FDG PET/CT imaging results with histological analysis collectively indicated a complete cure of the tumor-bearing rabbits at 10 or 14 days post-G-DEBs treatment. Therefore, day 14 post-treatment was considered the endpoint of the experiment. We did not collect the SUV_max_ values longer than 14 days post-treatment for prognostic information. Secondly, the sample size of the experiment on the VX2 tumor-bearing rabbit model was three in each group. An increase in the repeat number of each group may enhance the reliability and generalizability of the results. Thirdly, ^18^F-FDG is primarily excreted through the kidneys. The visualization of residual urine in the renal collecting system, along with physiological uptake and partial volume effects, can affect the observation of ^18^F-FDG uptake in renal tumors to some extent [36]. To overcome this limitation, a delayed ^18^F-FDG PET/CT scan after intravenous administration of furosemide and oral hydration is a useful method for improving the visualization of tumor uptake in the renal collecting system by decreasing the urinary artifact [37]. Finally, in terms of molecular mechanisms of the efficacy of G-DEBs, on the one hand, the injection of an embolic agent into the tumor-feeding arteries through DSA can lead to the death of tumor cells due to ischemia and hypoxia [38]. On the other hand, the locally released gemcitabine, a deoxycytidine analog, exerts antitumor effects by blocking the synthesis of DNA and interfering with the proliferation and survival of tumor cells [39]. The effect of tumor ischemia and the blockade of DNA synthesis will be evaluated to elucidate the mechanism of the combinatorial effect in a future study.

## 5. Conclusions

In this study, G-DEBs were successfully prepared with efficient embolization properties under DSA in rabbit VX2 renal tumors. TACE treatment with G-DEBs showed remarkable antitumor efficacy against rabbit VX2 renal tumors without obvious toxicity. Moreover, ^18^F-FDG PET/CT proved to be an effective and accurate method for evaluating and monitoring the therapeutic approach of TACE in rabbit VX2 renal tumors, which has great potential for clinical application in the future.

## Data Availability

The data presented in this study are available upon request from the corresponding author.
